# Efficient Detection of Nerve Agents through Carbon Nitride Quantum Dots: A DFT Approach

**DOI:** 10.3390/nano13020251

**Published:** 2023-01-06

**Authors:** Yasair S. S. Al-Faiyz, Sehrish Sarfaraz, Muhammad Yar, Sajida Munsif, Adnan Ali Khan, Bin Amin, Nadeem S. Sheikh, Khurshid Ayub

**Affiliations:** 1 Department of Chemistry, College of Science, King Faisal University, Al-Ahsa 31982, Saudi Arabia; 2 Department of Chemistry, COMSATS University Islamabad, Abbottabad Campus, Abbottabad 22060, Pakistan; 3 Centre for Computational Materials Science, University of Malakand, Chakdara 18800, Pakistan; 4 Department of Chemistry, University of Malakand, Chakdara 18800, Pakistan; 5 Department of Physics, Abbottabad University of Science & Technology, Abbottabad 22010, Pakistan; 6 Chemical Sciences, Faculty of Science, Universiti Brunei Darussalam, Jalan Tungku Link, Gadong BE1410, Brunei

**Keywords:** organophosphorus compounds, V-series nerve agents, frontier molecular orbital (FMO), electron density differences (EDD), sensing

## Abstract

V-series nerve agents are very lethal to health and cause the inactivation of acetylcholinesterase which leads to neuromuscular paralysis and, finally, death. Therefore, rapid detection and elimination of V-series nerve agents are very important. Herein, we have carried out a theoretical investigation of carbon nitride quantum dots (C_2_N) as an electrochemical sensor for the detection of V-series nerve agents, including VX, VS, VE, VG, and VM. Adsorption of V-series nerve agents on C_2_N quantum dots is explored at M05-2X/6-31++G(d,p) level of theory. The level of theory chosen is quite adequate in systems describing non-bonding interactions. The adsorption behavior of nerve agents is characterized by interaction energy, non-covalent interaction (NCI), Bader’s quantum theory of atoms in molecules (QTAIM), frontier molecular orbital (FMO), electron density difference (EDD), and charge transfer analysis. The computed adsorption energies of the studied complexes are in the range of −12.93 to −17.81 kcal/mol, which indicates the nerve agents are physiosorbed onto C_2_N surface through non-covalent interactions. The non-covalent interactions between V-series and C_2_N are confirmed through NCI and QTAIM analysis. EDD analysis is carried out to understand electron density shifting, which is further validated by natural bond orbital (NBO) analysis. FMO analysis is used to estimate the changes in energy gap of C_2_N on complexation through HOMO-LUMO energies. These findings suggest that C_2_N surface is highly selective toward VX, and it might be a promising candidate for the detection of V-series nerve agents.

## 1. Introduction

V-series nerve agents are lethal organophosphorus compounds that can inhibit acetylcholinesterase present in the central nervous system. The inactivation of acetylcholinesterase results in the accumulation of acetylcholine in the synapse, which can lead to neuromuscular paralysis and, finally, death [1,2,3]. These nerve agents display a potential threat to the community due to their physical properties (odorless, colorless, high volatility), higher toxicity, facile synthesis, and rapid action [4]. V-series nerve agents are devastating weapons used by terrorists due to their dispersive and highly lethal nature [5,6]. For the last two decades, V-type nerve agents have been used in various terrorist attacks [7,8,9,10,11].

V-series are considered more potent than G-series due to their greater resistance to detoxification and higher stability [12]. Therefore, quick, effective analytical tools are required to detect V-type nerve agents that will help to prevent terrorist attacks utilizing these agents. A variety of alternative techniques have been developed and used, such as gas chromatography along with mass spectrometry [13,14], fluorescence spectroscopy [15], ion mobility spectrometry (IMS), desorption electrospray ionization (DESI) [16], paper-based sensors [17], and membrane technology [18]. Although the mentioned techniques are desirable to capture and destroy harmful CWAs, these techniques are accompanied by some drawbacks such as complex procedures, user-unfriendly, and being expensive. In contrast, electrochemical sensors are most commonly employed for the detection of various analytes due to their rapid response, low cost, user-friendly nature, and simplicity [19].

Literature reveals that several types of materials have been investigated as electrochemical sensors for rapid and selective detection of nerve agents and other lethal CWA. For example, magnesium oxide nano-sheets, 3D graphene, boron nitrite nano-sheets, metal organic frameworks (MOFs) [20,21,22], covalent organic frameworks (COFs) [23,24], graphitic carbon nitride [25], robust hydrophobic MOFs [26], chemo sensors [27], and graphene-based composite sensors [28,29] have been used in this regard. These materials show many valuable properties in the detection and destruction of toxic pollutants and harmful CWAs. Changes in electronic properties upon adsorption of aromatic analytes (coronene, benzene, circumcirumcoronene, and circumcoronene) over Cu(111) and Au(111) are described well through interface dipole interactions [30,31]. However, these materials possess a small surface area, low porosity, rapid deactivation, less active sites for adsorption, and low reproducibility. Thus, a space is available to explore new sensing material, which must exhibit rapid response, large surface area, high sensitivity, and selectivity.

Herein, we designed and studied nitrogen-containing carbon nitride quantum dots (C_2_N) as a sensor for the detection of V-series nerve agents [32]. The C_2_N quantum dots have shown some remarkable properties, such as greater thermal stability, high electron-rich nitrogen content, and high surface area [33]. C_2_N shows higher specific surface area, good thermal stability, and conductivity than other CN-based compounds. According to the study of Tian et al., the limit of thermal stability of different material composites with C_2_N in different stoichiometry is ≈700–750 °C [34]. We previously showed through MD simulations that the structure of C_2_N is quite stable beyond 400 °C [35]. Many promising applications of C_2_N have been reported in the literature, such as fluorescence spectroscopy [36] and photocatalysis [37,38,39,40]. Similarly, C_2_N has been extensively explored theoretically for hydrogen storage [41], selective detection of picric acid [42], detection of ammonia [43], and batteries [44,45]. Recently, 2D C_2_N surface has been investigated for the selective detection of NH_3_ as an electrochemical sensor [43,46]. The presence of an electron-rich nitrogenated cavity makes the adsorption process facile; therefore, C_2_N surface is a promising candidate for adsorption and sensing applications [47].

In our previously reported studies on C_2_N surface, C_2_N showed high selectivity towards NH_3_ (among studied toxic analytes; HF, PH_3_, HCN, NH_3_, and H_2_S), NM (N-mustard) (among studied analytes; GA, GB, GF, GD, NM, and SM) and NI_3_ (among studied nitrogen halides; NBr_3_, NCl_3_, and NI_3_) on conductivity basis. In summary, the C_2_N surface has remained selective towards analytes that exhibit S–H, N–H, or C–H bonds of a methyl group attached to a phosphorus or nitrogen group [40,43,47,48]. Additionally, C_2_N surface bears a well-defined activity that is better suited for the adsorption of V-type nerve agents. An extensive literature survey reveals that literature is silent upon the evaluation of interactions of V-type nerve agents (VX, VE, VM, VR, VG) with C_2_N surface [49]. In the current study, we have carried out DFT calculations to study the mode of adsorption of five different V-series nerve agents on C_2_N surface. The perturbation in band gap upon complexation is evaluated through frontier molecular orbital (FMO) analysis. The nature of the interactions is examined by the quantum theory of atoms in molecules (QTAIM), non-covalent interaction (NCI), symmetry adapted perturbation theory (SAPT0) analysis, and electron density differences (EDD).

## 2. Computational Methodology

All optimizations were performed using the M05-2X method with a 6-31++G(d,p) level of theory, and calculations were computed via Gaussian 09 software. M05-2X, a calibrated hybrid method with two non-local exchange functions, is best for capturing non-covalent interactions [50]. We used the M05-2X functional for our DFT study based on already reported benchmark studies in the literature. This functional is best specifically when dispersion interactions dominate among non-covalent interactions, as in the case of our study. A benchmark study was performed by Burns et al. on several hybrid functionals, and they reported that dispersion dominated non-covalent interactions could be better studied through the M05-2x hybrid functional [51]. Quite rich literature is available on the efficiency of the M05-2X hybrid functional for non-bonding interactions [25,52,53,54,55,56,57]. While frontier molecular orbitals (FMO) analysis was performed using the B3LYP method with 6-31G(d) level of theory because the accuracy of this method is well reported in the literature [58,59,60]. Visualization of the optimized geometries was performed through GaussView 5.0 and Chemcraft software [61,62]. Several possible orientations for each analyte were studied to obtain the most stable configuration with the lowest energy of each complex. The interaction energies of interacting fragments were evaluated by the following equation:

**ΔE = E_complex_ − (E_V-series_ + E_C2N_)** (1)

In Equation (1): E_complex_, E_C2N_, and E_V-series_ represent the electronic energies of V-series nerve agents C_2_N complexes, isolated C_2_N surface, and V-series nerve agents, respectively. Frontier molecular orbital (FMO) analysis is carried out to analyze the change in the energy band gap [63]. To study the charge transfer between V-series nerve agents and C_2_N surface, natural bond orbital (NBO) and electron density differences (EDD) analysis was performed [64]. EDD analysis provides a visual portrayal of the charge transfer among interacting fragments through colored isosurfaces and further validates NBO results [65].

NCI analysis is computed to visualize and differentiate the interactive forces existing among the analyte and C_2_N surface. Multiwfn 3.7 software is employed to construct 2D RDG graphs and 3D isosurfaces of V-series@C_2_N complexes. The non-covalent interaction index is generally dependent on two variables, RDG (reduced density gradient) and ρ (electron density), and they are interrelated by the equation given below:

(2) $$RDG=\frac{1}{2{\left(3\pi \right)}^{1/3}}\;\frac{\nabla \rho }{\rho^{3/4}}$$

According to the divergence theorem, the density of net gradient flux is indicated by the Laplacian (∇^2^ρ) sign. A positive sign of Laplacian (∇^2^ρ > 0) indicates that the flux is leaving, whereas the negative sign (∇^2^ρ < 0) shows that flux is entering in an infinitesimally small volume in the vicinity of the reference point [47]. Therefore, the sign of Laplacian indicates the accumulation or depletion of electron density at the reference point. In NCI analysis, the nature of interactions is described via a color scheme dependent on the value of sign(λ_2_)*ρ*. The appearance of blue spikes and isosurfaces show strong electrostatic interactions with a corresponding negative value of sign(λ_2_)*ρ* in 2D-RDG graphs and 3D plots, respectively. While sign(λ_2_)*ρ* with small negative values indicates weak van der Waals forces, appearing as green spikes and isosurfaces in 2D-RDG graphs and 3D plots, respectively. Red isosurfaces appear when sign(λ_2_)*ρ* has large positive values and indicates repulsive interactions [42,56,65].

Symmetry-adapted perturbation theory (SAPT) analysis is also carried out to understand the mode of interaction present between the V-series and C_2_N surface. SAPT0 calculations were computed with PSI4 software. SAPT0 analysis consists of four components of interaction energies, i.e., induction (ΔE_ind_), dispersion (ΔE_disp_), electrostatic (ΔE_elstat_), and exchange (ΔE_exch_). The equation for SAPT0 ΔE_int_ can be written as:

**ΔE_int_ = ΔE_ind_ + ΔE_disp_ + ΔE_elstat_ + ΔE_exch_** (3)

Hence, SAPT0 total consists of the sum of these four contributing factors, i.e., ΔE_ind_, ΔE_disp_, ΔE_elstat_, and ΔE_exch_, which stabilizes V-series nerve agents with C_2_N upon complexation [66]. Among these components, ΔE_disp_, ΔE_elstat_, and ΔE_ind_ are attractive forces, whereas the ΔE_exch_ factor is a repulsive force.

Another useful tool for quantifying non-covalent interactions is QTAIM analysis. Important topological parameters, including electronic density (ρ), Laplacian (∇^2^ρ), and total energy density (H(r)), are employed to confirm the types of interactions via bond critical points (BCPs). Similarly, other parameters, such as the local potential energy V(r), local Lagrangian kinetic energy G(r), and total energy density H(r), are being used to understand the types of interactions existing between the V-series and C_2_N surface. Whereas, in topological analysis, Laplacian (∇^2^ρ) and electronic density (ρ) are the two main parameters used to examine the strength of a bond for particular BCPs [42,67,68,69]. Overall, we used Multiwfn, VMD, Gnuplot, and GIMP for NCI, QTAIM, and EDD analyses, while the SAPT0 graph was generated through MS excel. The features of PCs used are Workstation Intel Core i7 6700 K processor, 8 MB Cache 6th Generation Gigabit H 110 main board, with DDR IV, RAM 1900 MHZ, 16 GB, tower casing 400-Watt supply, DVD RW, SATA 10,000 GB hard disk. The average time taken for optimization in this study is two days, 19 h 7 min, while for properties average run time is 5 h 11 min.

## 3. Results and Discussion

### 3.1. Geometric Optimization and Adsorption Energies

**Figure 1:**
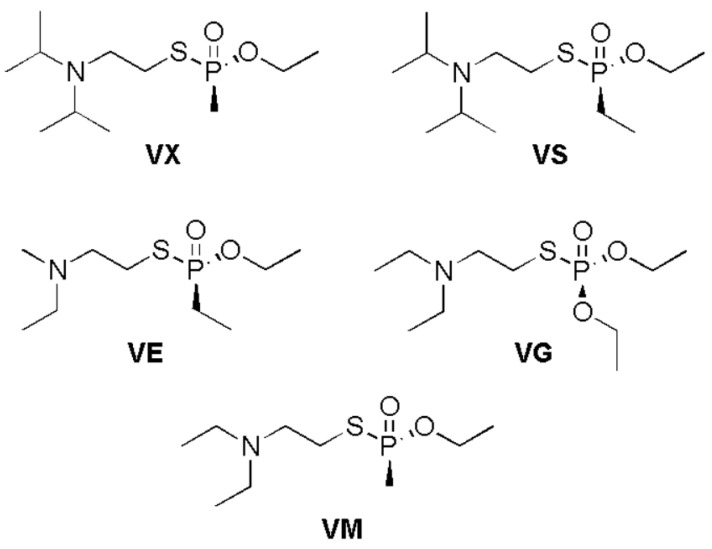
Chemical structures of studies V-type nerve agents.

In the current study, the adsorption behavior of five different V-series nerve agents was examined against C_2_N surface. The chemical structures of these V-series nerve agents are presented in Figure 1. V-series nerve agents selected as analytes are **VX**; O-Ethyl-S-[2(diisopropylamino)ethyl] methylphosphonothioate, **VS**; O-Ethyl-S-[2(diisopropylamino)ethyl] ethylphosphonothioate **VE**; O-Ethyl-S-[2-(diethylamino)ethyl] ethylphosphonothioate. **VG**; O,O-Diethyl-S-[2-(diethylamino)ethyl] phosphorothioate and **VM**; O-Ethyl-S-[2-(diethylamino)ethyl] methylphosphonothioate (Figure 1). In V-type nerve agents, a thiophosphate (S-P=O) group makes up the central unit. On one side of the thiophosphate group, an -OR group is attached, while on the other side, R group is present (the R group could be methyl or ethyl) (see Figure 1) [70].

**Figure 2:**
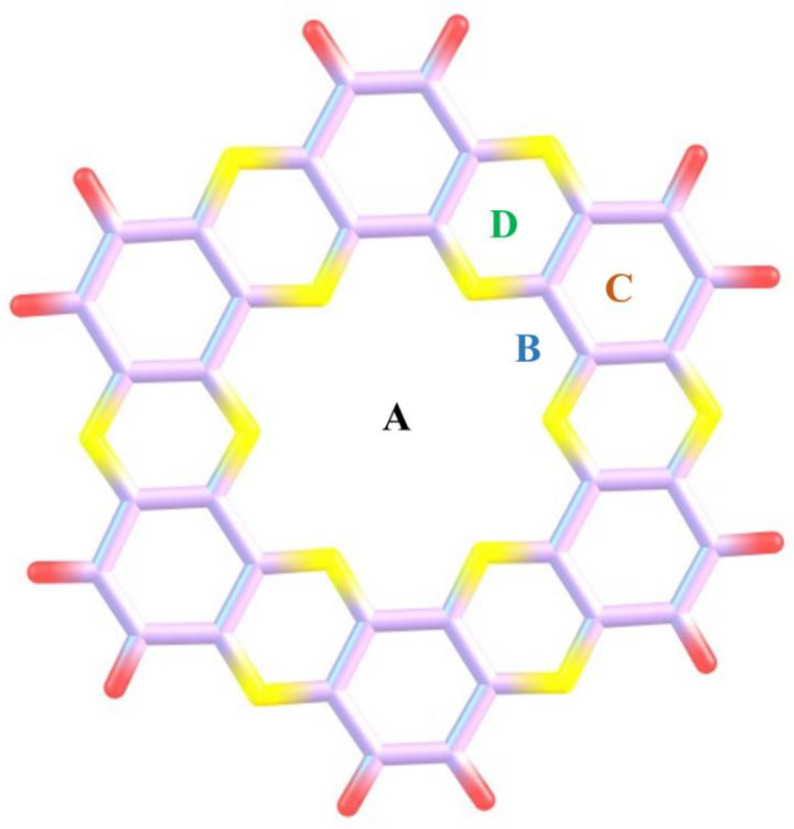
Optimized geometry of C_2_N surface with four binding sites (A, B, C, and D) at the M05-2X/6-31++G(d,p) level of theory (yellow shows nitrogen, red represents hydrogen and light purple represents carbon).

The optimized C_2_N quantum dot is shown in Figure 2. C_2_N consists of alternate pyrazine and benzene rings. In C_2_N material, the presence of an electron-rich nitrogenated cavity might help in the adsorption of analytes and thus make them suitable candidates for sensing studies against CWAs. The C_2_N surface consists of four appropriate binding sites; **A**; the center of C_2_N surface, **B**; triangular region made by C-N atoms, **C**; at the top of benzene rings and **D**; is over pyrazine rings (see Figure 2) [71,72].

**Figure 3:**
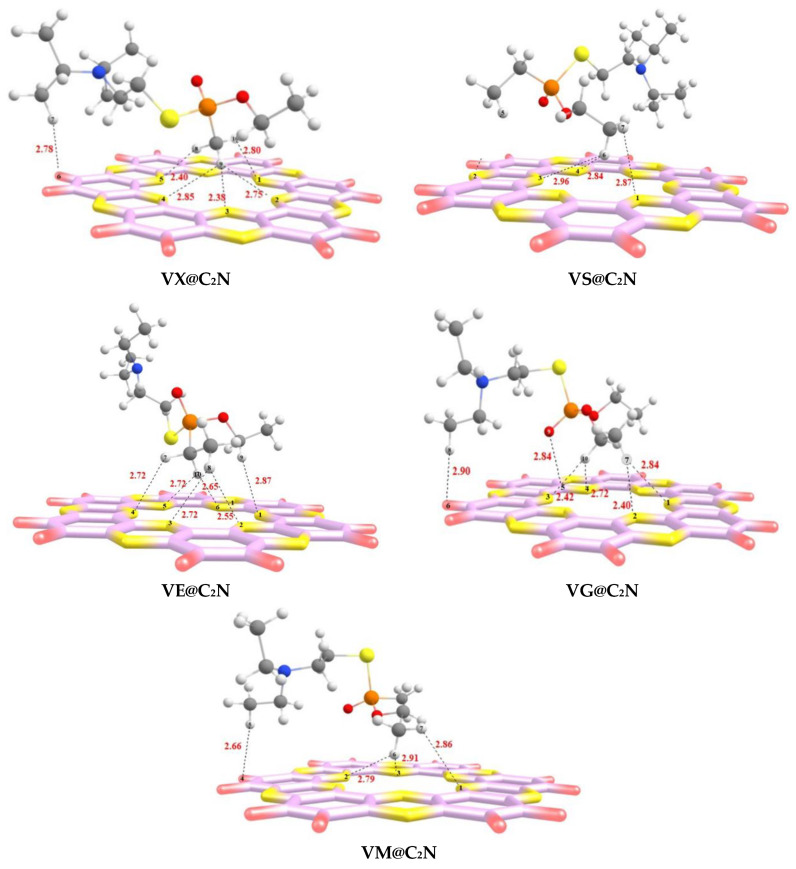
The optimized geometries of V-series@C_2_N complexes at M05-2X/6-31++G(d,p) level of theory.

All possible orientations were explored over four favorable binding sites to get the most stable and lowest energy configuration for all V-series@C_2_N complexes. The most stable optimized geometries of V-series@C_2_N complexes are presented in Figure 3.

Interaction distance and energy (E_int_) are two important parameters for real estimation of interaction behavior [73]. The bond interaction distance decreases when the analyte approaches the surface (C_2_N) for complexation. Interactions existing between V-series nerve agents and C_2_N surface are attributed to the minimum interaction distance, which can be understood via interaction energy. We have presented interaction distances for all complexes (V-series@C_2_N) along with corresponding interaction energy in Table 1.

The interaction energies of VG@C_2_N, VM@C_2_N, and VX@C_2_N complexes are quite comparable, and interaction energy values are −17.81 kcal/mol, −17.69 kcal/mol, and −17.64 kcal/mol, respectively (see Table 1). Whereas the other two complexes, i.e., VE@C_2_N and VS@C_2_N, have interaction energy values of −13.97 kcal/mol and −12.93 kcal/mol, respectively. The optimized stable geometry of **VX@C2N** showed strong non-covalent interactions between the H-atoms of **VX** and the N-atoms of **C_2_N**. The strongest H---N interactions are observed between H9---N3 and H8---N5 atoms (see Figure 3) of the **VX@C_2_N** complex with interaction distances of 2.38 Å and 2.40 Å, respectively. Whereas relatively weak H---N interactions are observed between H10---N1, H9---N2, and H9---N4 atoms of **VX@C_2_N** complex with interaction distances of 2.75 Å, 2.80 Å, and 2.85 Å, respectively. Moreover, van der Waals interactions are also observed between the H7 atom of VX and the H6 atom of C_2_N with an interaction distance of 2.78 Å (see Figure 3).

The most stable configuration of **VS@C_2_N** shows that H-atoms of the ethyl group of **VS** are projecting towards the C_2_N surface and interacting with N-atoms of the C_2_N cavity. The bond distances of H---N interactions range from 2.84 Å to 2.99 Å in the **VS@C_2_N** complex. On the other hand, the stable geometry of the **VE@C_2_N** complex shows the least interaction distance of 2.55 Å for H8---N2 atoms. In this case, the H-atoms of **VE** are inclined towards **C_2_N** surface, thus interacting with the N-atoms of **C_2_N.**

Similarly, the most stable configuration of the **VG@C_2_N** complex was obtained after optimizing several interactive modes on **C_2_N** surface. The stable geometry of the **VG@C_2_N** complex reveals that the H-atoms of **VG** interact with the N-atoms of the surface. H10 and H11 atoms of VG both show strong interactions with N-atoms of **C_2_N,** i.e., H10---N3, H10---N4, H7---N1, and H7---N2 with interaction distances of 2.42 Å, 2.72 Å, 2.84 Å, and 2.40 Å, respectively. **VG@C_2_N** complex also shows interaction distances of 2.84 Å (O9---C5) and 2.90 Å (H8---H6).

In the case of the **VM@C_2_N** complex, the H-atoms of the ethyl group of **VM** were heading towards the N-atom’s surface, whereas the remaining part of the **VM** analyte flipped away from the surface (C_2_N), giving an umbrella-like appearance. Strong H---N interactions are observed at interaction distances of 2.86, 2.79, and 2.913 Å for H7---N1, H6---N2, and H6---N3, respectively, between the H-atoms of **VR** and the six nitrogen atoms of the C_2_N cavity. One weak van der Waals interaction is observed at a bond distance of 2.66 Å (H5---H4).

For all V-series@C_2_N, the interaction energy values reveal that all analytes are physiosorbed on C_2_N surface. The strongest interaction from studied V-series nerve agents is observed for **VG** nerve agents with an interaction energy of −17.81 kcal/mol. The interaction energy trend for studied V-series nerve agents is **VG@C2N > VM@C2N > VX@C2N > VE@C2N > VS@C2N**.

In the case of **VS@C_2_N** and **VE@C_2_N** complexes, the longer bond distance between analytes and C_2_N atoms resulted in a lowering of interaction energy after complexation as compared to the rest of the studied V-series nerve agents.

### 3.2. Non-Covalent Interactions (NCI) Analysis

NCI analysis is a useful tool for characterizing the nature of interactions, and it helps in differentiating between different nonbonding interactions, such as weak van der Waals interactions, H-bonding, and repulsive interactions. The NCI analysis gives a clear explanation of non-covalent interactions based on the second eigen value with the first differential of electron density (*ρ*), known as RDG (reduced density gradient) [74]. In NCI analysis, the 2D-RDG graphs are observed by plotting the reduced density gradient (RDG) at the y-axis versus signλ2(*ρ*) at the x-axis. In signλ2(*ρ*), *ρ* indicates the bonding strength, whereas signλ2 gives the information regarding the type of bonding. For repulsion, the positive value of signλ2(*ρ*) indicates the presence of weak van der Waals interactions. The color map in the 3D isosurface is also dependent on the value of signλ2(*ρ*) of NCI analysis. If the values are positive, i.e., signλ2(*ρ*) > 0, red isosurfaces result, indicating steric repulsive interaction. Negative values, i.e., signλ2(*ρ*) < 0, resulted in the appearance of green isosurfaces, which represent van der Waals interactions. The appearance of blue isosurfaces in 3D images among interacting fragments of complex resulted from a large negative value of signλ2(*ρ*), i.e., signλ2(*ρ*) > −0.02, which corresponds to strong electrostatic interactions [69].

**Figure 4:**
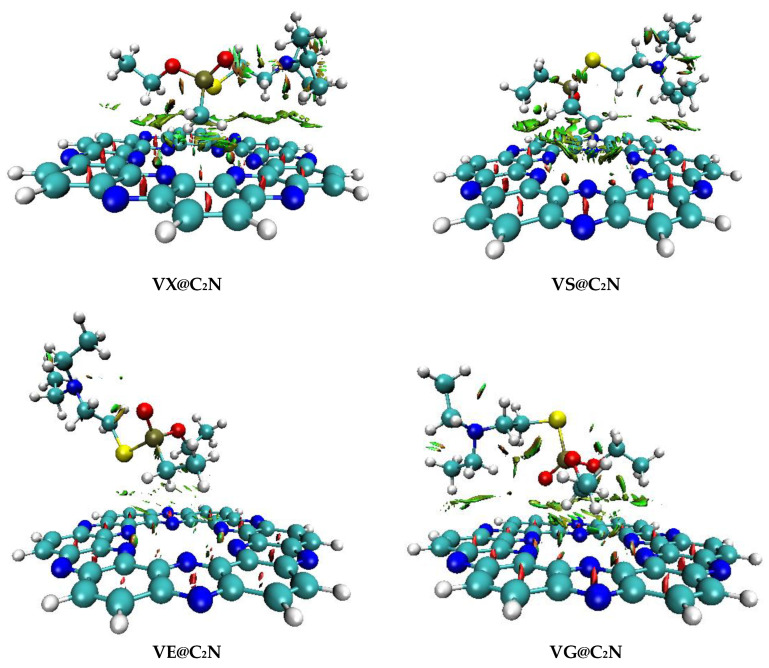

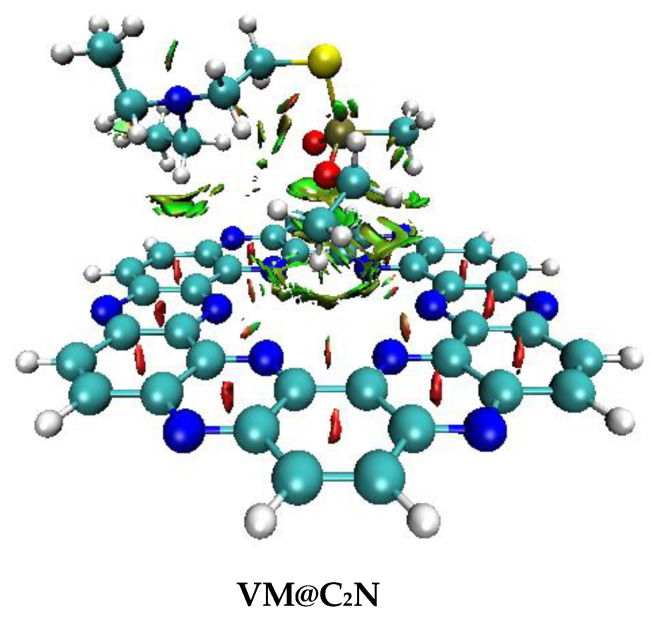
NCI 3D isosurfaces of optimized geometries of stable V-series@C_2_N complexes computed at M05-2X method (iso value 0.05 a.u.) here red color is for repulsive interaction, green color indicates weak van der Waal’s forces interactions, and blue color is for strong electrostatic interactions.

**Figure 5:**
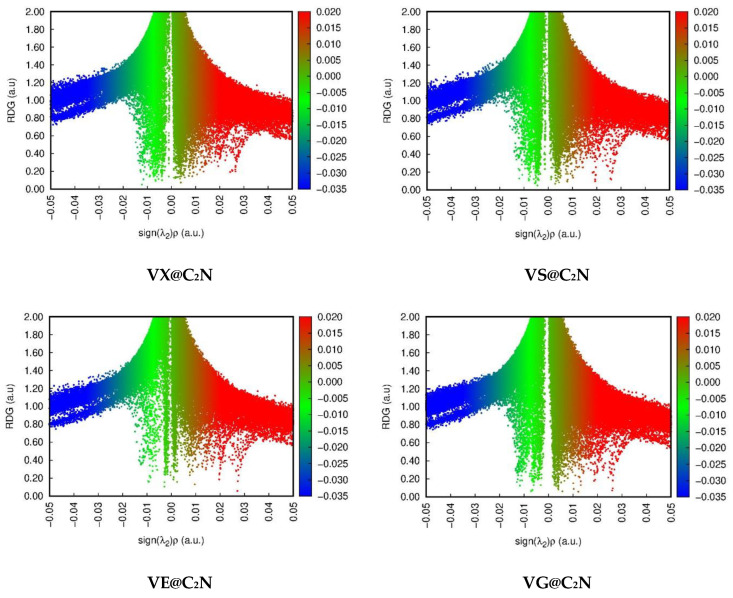

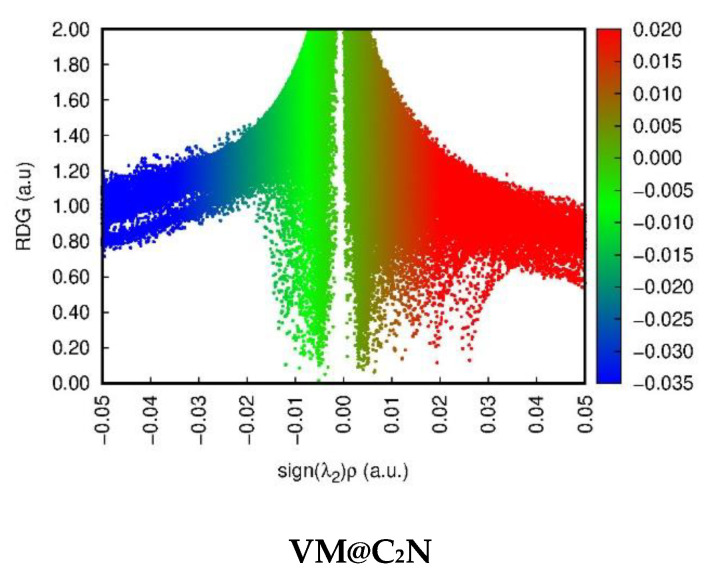
2D-RDG plots of studied complexes via NCI analysis.

The 2D-RDG graphs and colored 3D isosurfaces are presented in Figure 4 and Figure 5, respectively. In NCI analysis, the colored map reveals the appearance of a green isosurface between C2N surface and V-series nerve agents, which indicates the existence of weak van der Waals interactions. The 3D green isosurfaces in the case of VG@C_2_N, VM@C_2_N, and VX@C_2_N complexes are more prominent as compared to the other two complexes, which shows the stability of these complexes, which is in accordance with the results of interactions energy. Furthermore, 2D-RDG plots depict that the projection of scattered green spikes in all V-series@ C_2_N complexes appears in the range of 0.00 a.u. to −0.015 a.u., which confirms weak van der Waals interactions in all complexes. The existence of steric repulsion in all complexes is also confirmed through red-colored 3D isosurfaces, which are observed in the center of pyrazine and benzene rings of the C_2_N unit (Figure 4).

Similarly, more negative values of λ_2_ and deep RDG confirms the presence of strong electrostatic interactions, specifically hydrogen bonding. 2D NCI graphs depict that the spikes appear at signλ2(*ρ*) > −0.01 (a.u.). which presents strong electrostatic interactions, whereas, below this negative value, London dispersion force exists.

### 3.3. Quantum Theory of Atoms in Molecules (QTAIM) Analysis

The non-covalent interactions among V-series analytes and C_2_N surface are further explored via Bader’s quantum theory of atoms in molecules (QTAIM) analysis. The topological parameters employed to study the nature of non-covalent interactions at BCPs are electronic density ρ(r), Laplacian of electronic density ∇^2^ρ(r), local Potential energy V(r), local Lagrangian kinetic energy G(r), and total energy density H(r). For covalent bonds, values of ρ must be positive and greater than 0.1 a.u., while Laplacian (∇^2^ρ) is always a large value with a negative sign. On the contrary, for non-covalent interactions, the values of ρ are always less than 0.1 a.u. (ρ < 0.1 a.u.) and ∇^2^ρ is positive with small values [75,76]. Similarly, for covalent interactions, the ratio of −V/G > 2, whereas for non-covalent interactions, the ratio of −V/G must be less than 1 (−V/G < 1) [77].

**Figure 6:**
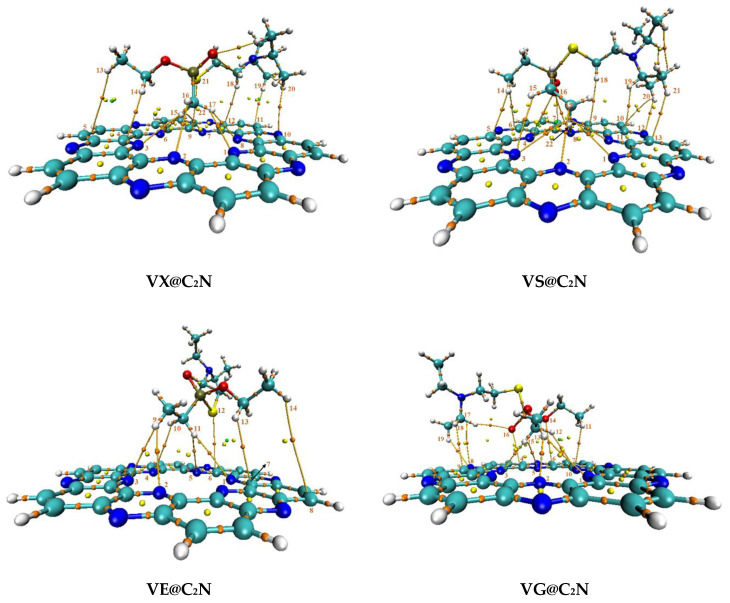

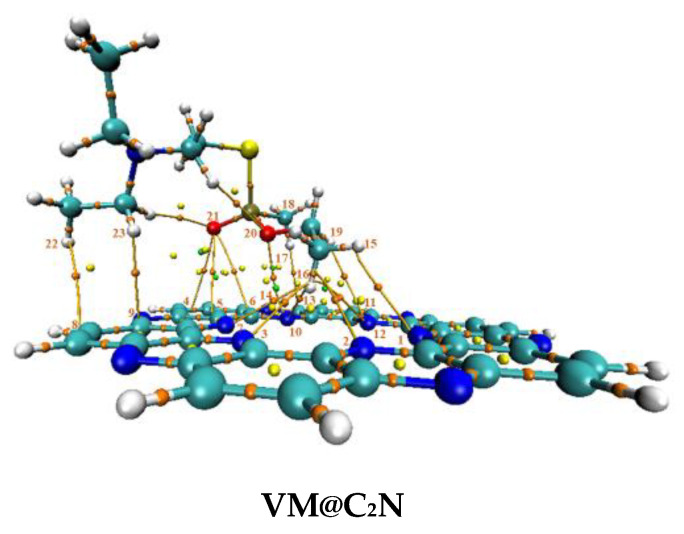
QTAIM analysis of studied V-series@C_2_N complexes and yellow lines between nerve agent molecules and C_2_N sheet indicates bond paths, whereas orange-colored dots present on bond paths show the bond critical points (BCPs).

Geometries of all complexes are optimized at M05-2X/6-31++G(d,p) level of theory to characterize QTAIM results. The results of topological parameters calculated via QTAIM analysis for V-series@C_2_N complexes are reported in Table 2. Topological 3D isosurfaces of all studied V-series@C_2_N complexes obtained through QTAIM analysis are shown in Figure 6. The values of electron density (ρ) and Laplacian (∇^2^ρ) reported in Table 2 justify the existence of non-covalent interactions in all studied V-series@C_2_N complexes. A total of 11 BCPs are observed in the case of the VX@C_2_N complex with six H---N, four H---C, and one S---C bond interactions. The values of electron density (ρ) and Laplacian (∇^2^ρ) are in the range of 0.004 to 0.012 a.u. and 0.011 to 0.037 a.u., respectively, which clearly indicates the existence of non-covalent weak interactions. The values of total energy density H(r), local Potential energy V(r), and local Lagrangian kinetic energy G(r) are also observed in the range of non-covalent weak interactions. The maximum number of BCPs are observed for the VS@C_2_N complex, which is 14 (see Table 2). The values of electron density ρ for observed BCPs is in the range of 0.003 to 0.009 a.u., and the range of values for Laplacian ∇^2^ρ is 0.012 to 0.026 a.u.

Similarly, six BCPs are present for the VE@C_2_N complex with five H---N and one S---C bond interactions. The strongest interactions are obtained for H11---N1 and H11---N5 bonds with electron density ρ and Laplacian ∇^2^ρ values of 0.003 a.u. 0.010 a.u., respectively. In the case of the VG@C_2_N complex, 11 BCPs are observed with seven H---N, two H---C, and two O---C bond interactions. The values of electron density (ρ) and Laplacian (∇^2^ρ) are in the range of 0.004 to 0.011 a.u. and 0.014 to 0.036 a.u., respectively. For the VM@C_2_N complex, the total BCPs observed are 13, with seven H---N, three O---C, two H---C, and one O---N bond interaction (see Figure 6 & Table 2). The values of electron density (ρ) and Laplacian (∇^2^ρ) are observed in the range of 0.004 to 0.009 a.u. and 0.014 to 0.029 a.u., respectively, which indicate that only non-covalent interactions exist between VM analyte and C_2_N surface.

The ratio of −V/G is also calculated for each BCPs of all studied V-series@C_2_N complexes. The highest individual −V/G values for studied complexes i.e., **VX@C2N, VS@C2N, VE@C2N, VG@C2N** and **VM@C2N** are 0.96, 0.96, 0.70. 0.91 and 0.86, respectively (Table 2). Moreover, the ratio of −V/G also indicates that the local potential energy V(r) parameter is dominant in all V-series@C_2_N complexes. Potential energy mainly rises due to the rise in values of Laplacian and electron density. The values of Laplacian, electron density, and −V/G also indicate that non-covalent interactions exist in all studied complexes.

Apparently, the observed values of all topological parameters, i.e., electron density ρ(r), Laplacian ∇^2^ρ(r) total energy density H(r), Potential energy V(r), and Lagrangian kinetic energy G(r) for all studied V-series@C_2_N complexes indicate that only non-covalent interactions exist among nerve agents and 2D surface. Therefore, all the V-series nerve agents are physiosorbed on **C_2_N** surface. Among studied V-series nerve agents, the lowest BCPs are examined in the case of the VE@C_2_N complex, i.e., six. Furthermore, the topological parameters such as ρ, ∇^2^ρ, G, V, and H have the lowest values in cases of VE@C_2_N complex as compared to the rest of V-series@C_2_N complexes.

### 3.4. SAPT0 Analysis

**Figure 7:**
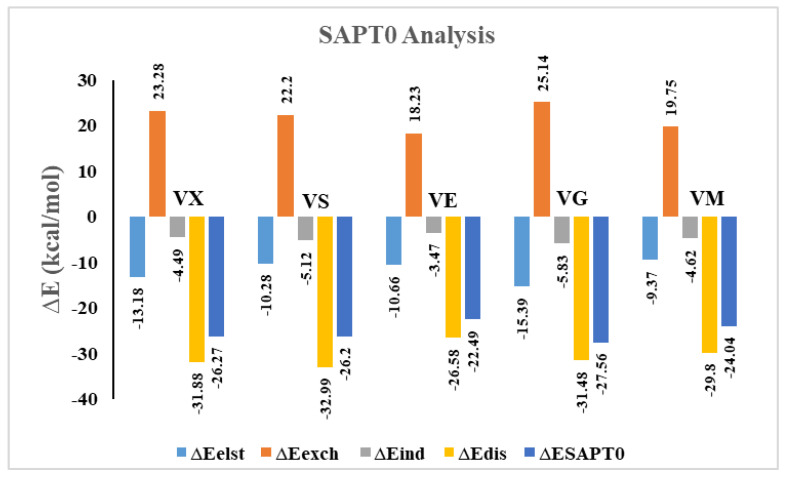
SAPT0 analysis of V-series@C_2_N complexes graphical representation.

Symmetry adapted perturbation theory (SAPT0) analysis is used to characterize the interactions quantitatively between V-series nerve agents and C_2_N surface. SAPT0 analysis consists of four contributing factors (interaction energies), i.e., electrostatic (ΔE_elst_), exchange (ΔE_exch_), induction (ΔE_ind_), and dispersion (ΔE_disp_) [78]. The sum of these four components, E_elst_, E_exch_, E_ind_, and E_dis,_ gives the total SAPT0 energy (E_SAPT0_) [79]. The contribution of each interaction energy component is obtained through SAPT0 analysis, and values are reported in Table 3, whereas a graphical representation of V-series@C_2_N complexes is shown in Figure 7.

The highest contribution of the E_exch_ component is observed in the VG@C_2_N complex (25.14 kcal/mol), followed by VX@C_2_N, VS@C_2_N, VM@C_2_N, and VE@C_2_N complexes with E_exch_ values of 23.28, 22.20, 19.75, and 18.23 kcal/mol, respectively. The contribution of remaining energy components for the VX@C_2_N complex are −13.18 kcal/mol (E_elst_), −4.49 kcal/mol (E_ind_), and −31.88 kcal/mol (E_disp_). SAPT0 results clearly indicate that the dispersion component is the major stabilizing factor (64.34%), followed by the E_elst_ component, which contributes 26.60%. Similarly, for the VS@C_2_N complex, the values of E_elst_, E_disp,_ and E_ind_ are −10.28, −32.99, and −5.12 kcal/mol, respectively. In the case of the VE@C_2_N complex, again, E_disp_ is a dominant contributing factor with 65.29% (−26.58 kcal/mol) contribution. Whereas E_elst_ and E_ind_ are less dominant towards total SAPT0 with 26.18% (−10.66 kcal/mol) and 8.52% (−3.47 kcal/mol) contribution, respectively. VM@C_2_N complex again follows the same trend of SAPT energy components, where the E_disp_ factor shows the highest contribution (68.05%), while E_elest_ and E_ind_ indicate less contribution of 21.39% and 10.55% towards total SAPT0, respectively.

SAPT0 analysis indicates that the majority of SAPT0 energy components are negative, which reveals that attractive forces are more dominating between analytes and C_2_N surface (see Table 3). Whereas the exchange component (∆E_exch_) shows positive values, which reveals the presence of repulsive force between two interacting components. The highest stabilization energy is observed in the case of the VG@C_2_N complex among E_SAPT0_ energy values, which is in accordance with interaction energy (E_int_) analysis (see Table 1). The overall order of the SAPT0 component’s contribution towards total SAPT0 energy (E_SAPT0_) is E_disp_ > E_elest_ > E_ind_. This trend indicates that the major stabilizing factor among SAPT0 components is E_disp_.

## 4. Electronic Properties

### 4.1. Natural Bond Orbital (NBO) Analysis

The NBO analysis is carried out to analyze the quantity of charge transfer after the complexation of V-series nerve agents with C_2_N surface. Electronic properties play an important role in understanding the nature of interactions existing among nerve agents and C_2_N surface. The values of NBO charges upon the interaction of analytes and surface are given in Table 3. The negative value of NBO charges shows that the charge is transferred from surface to analyte and vice versa [80].

NBO analysis reveals that the values of NBO charges appeared in the range of −0.023 e^−^ to −0.002 e^−^. Here the negative sign indicates that, for all V-series@C_2_N complexes, the charge is transferred from C_2_N surface to V-series agents. The NBO charge values of studied nerve agents are −0.023 e^−^, −0.020 e^−^, −0.013 e^−^, −0.012 e^−^, and −0.002 e^−^ for VX, VS, VE, VG, and VM, respectively (Table 4). This trend is observed due to charge transfer upon the interaction of the positively charged H-atoms of nerve agents (VX, VS, VE, VG, and VM) with the electron-rich C_2_N surface. The highest amount of charge transfer (−0.023 e^−^ and −0.020 e^−^) was examined for VX@C_2_N and VS@C_2_N complexes, respectively, which might be due to the strong electrostatic interaction (hydrogen bonding) of H-atoms with N-atoms of C_2_N cavity. Furthermore, small NBO charge transfer in the London dispersion force case of VM@C_2_N complex reveals the existence of weaker non-covalent interactions.

### 4.2. Electron Density Differences (EDD)

The type of interactions upon adsorption of nerve agents on C_2_N surface was further characterized through Electron Density Difference (EDD) analysis. EDD plots of V-series@C_2_N complexes (Isovalue = 0.004 a.u.) are given in Figure 7. The isosurfaces were obtained using multiwfn 3.7 software(Tian Lu, Beijing Kein Research Center for Natural Sciences). Electron density difference was calculated through the variance of electron density among V-series@C_2_N complexes and the aggregate electron density of bare C_2_N surface and isolated V-series nerve agents.

**Figure 8:**
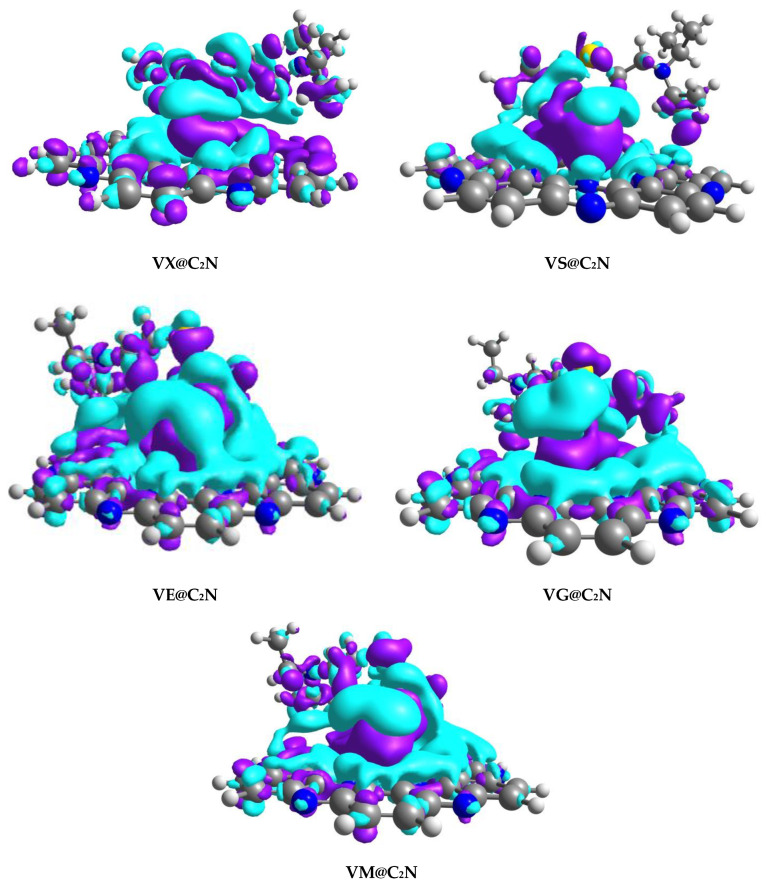
EDD plots of V-series@C_2_N with Iso value = 0.004 a.u. (Blue isosurfaces show accumulation of electron density, while purple isosurfaces represent depletion of electron density).

The appearance of blue and purple isosurfaces in EDD analysis indicates orbital interaction between C_2_N surface and nerve agents (Figure 8). Blue isosurfaces depict an accumulation of electronic density, whereas purple isosurfaces show a depletion of electron density. Blue isosurfaces appeared due to the electrostatic interaction of H-atoms of considered V-type nerve agents and N-atoms of C_2_N surface, which resulted in a higher accumulation of electron density among nerve agents and C_2_N surface. Moreover, blue surfaces also reveal sigma (σ) donation of charge from N-atoms of C_2_N surface to V-type nerve agents, thereby confirming charge transfer from surface towards analytes. The appearance of purple isosurfaces has also been noticed in Figure 8, which indicates the reduction of electron density at the interacting H-atoms of V-series agents. EDD plots revealed electronic density shifting among analytes and surface rings C_2_N, i.e., benzene and pyrazine rings. Electron Density Difference (EDD) results are also validated through charge transfer (NBO) analysis.

### 4.3. Frontier Molecular Orbital (FMO) Analysis

Upon complexation with V-series nerve agents, the variation in electronic properties of C_2_N sheet is evaluated via frontier molecular orbitals analysis. FMO analysis was employed to study the conductivity of the considered material through a change in band gap. Generally, conductivity rises with the decrease in the energy gap (HOMO-LUMO gap) and vice versa [81].

**Figure 9:**
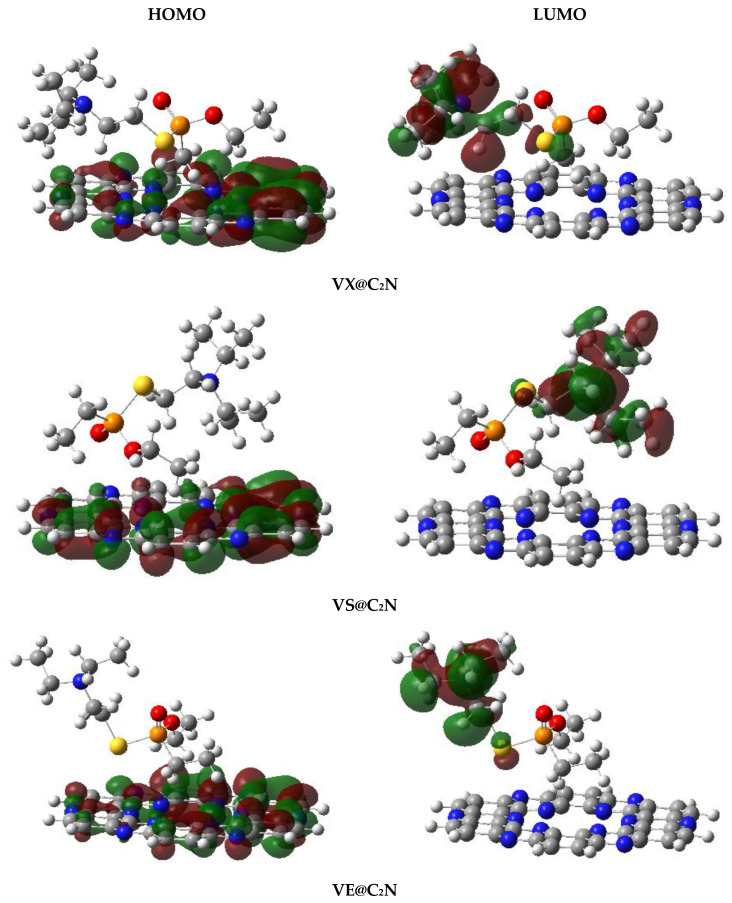

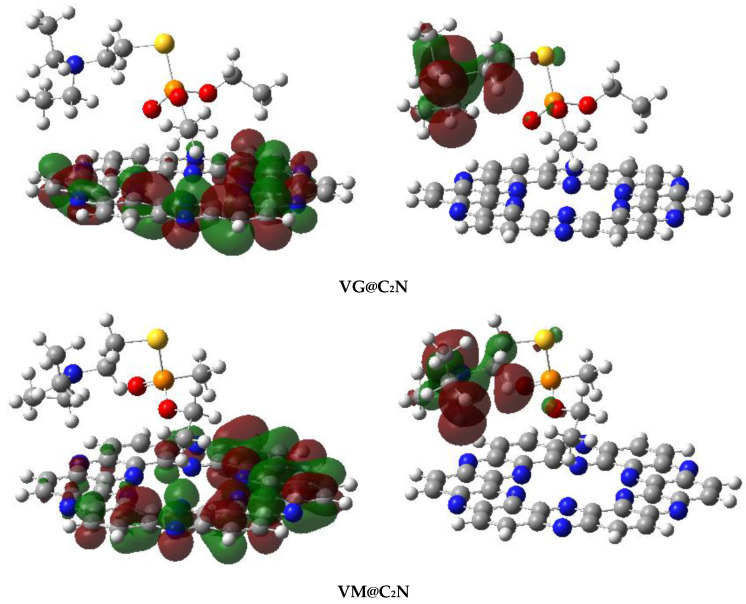
HOMO-LUMO densities of the studied V-series@C_2_N surface.

The values of HOMO-LUMO energies in a.u. and eV and their differences (band gap) in eV are reported in Table 4. Isosurfaces of HOMO and LUMO for studied complexes are presented in Figure 9. For bare C_2_N surface, the HOMO energy is −6.40 eV, the LUMO energy is −2.69 eV, and the energy gap (E_H-L_) for bare C_2_N surface is **3.71** eV. The HOMO-LUMO energy gaps (E_H-L_) of V-series@C_2_N complexes are **2.13** eV (VX@C2N), **2.63** eV (VS@C2N), **2.47** eV (VE@C2N), **2.81** eV (VG@C2N), and **2.80** eV (VM@C2N). For all studied V-series nerve agents, an appreciable reduction in the HOMO-LUMO energy gap (E_H-L_) is observed upon the adsorption of V-series analytes on C_2_N surface.

As a result of the complexation of C_2_N surface with analyte VX, the HOMO energy increases from −6.40 eV to −5.02 eV, and LUMO energy reduces from −2.69 eV to −2.89. Moreover, the HOMO-LUMO energy gap (E_H-L_) is changed from 3.71 eV to 2.13 eV. Similar behavior with slight differences is observed for the rest of the V-series nerve agents in terms of variation in energies of HOMO and LUMO and energy gap (E_H-L_). Upon adsorption of VS over C_2_N, the energy of HOMO increases (−5.37 eV), which results in a reduced E_H-L_ gap of 2.63 eV, compared to 3.71 eV for isolated C_2_N. Similarly, the interaction of VE with C_2_N surface increases the HOMO energy value and decreases LUMO energy; E_H-L_ is reduced to 2.47 eV (see Table 4). Moreover, VG and VM nerve agents also show the same trend upon complexation; HOMO energy values increase to −5.56 eV and −5.57, and LUMO energy values reduce to −2.75 eV and −2.78 eV, respectively. The E_H-L_ are also reduced to 2.81 eV and 2.80 eV for VG and VM complexes, respectively. Among studied V-series@C_2_N, the most prominent HOMO-LUMO energy gap (E_H-L_) reduction (2.13 eV) is observed for the VX@C_2_N complex.

In all studied V-series@C_2_N complexes, HOMO orbital density is located over C_2_N surface, whereas, in the case of LUMO, the orbital density is mostly distributed on V-series nerve agents (analytes). Overall, FMO data clearly displays that all studied V-series nerve agents show an appreciable reduction in the E_H-L_ gap upon complexation, which revealed that C_2_N surface shows higher sensitivity towards V-series nerve agents.

## 5. Recovery Time

The stability of an ideal sensor can be measured through its ability to reprocess. Suitable recovery time is essential for the adsorption of nerve agents because the very high recovery response of a sensor leads to poisoning of the surface, whereas a very short recovery time does not provide appreciable time for the analyte to stay on the surface. The recovery response time of a sensor is theoretically calculated via transition theory which is given by the equation:

(4) $$\tau =\upsilon^{-1}\exp\left(\frac{-E_{ads}}{KT}\right)$$

where τ, υ, K, E_ads_, and T represent recovery time, attempt frequency, Boltzmann constant interaction energy, and temperature of recovery, respectively. To evaluate the recovery time of C_2_N, an attempt frequency of 10^12^ s^−1^ is applied [82,83,84]. The recovery times (τ) of V-series@C_2_N complexes are calculated by using Equation (4) at three different temperatures, i.e., 298, 350, and 400 K. Recovery times of 8.53 s, 11.40 s, and 9.29 s are observed in the case of VX@C_2_N, VG@C_2_N, and VM@C_2_N complexes, respectively. Recovery time improves by rising temperature, i.e., the obtained values of recovery time at 400 K are 4.30 × 10^−3^ s, 5.33 × 10^−3^ s, and 4.58 × 10^−3^ for VX@C_2_N, VG@C_2_N, and VM@C_2_N complexes, respectively. However, the recovery time results seem much better compared to reported values for other surfaces. A short recovery time of 0.63 × 10^−6^ s at room temperature for the desorption of G-series nerve agent (GF) from graphdiyne (GDY) surface has been reported in the literature [85]. Moreover, for the desorption of NO_2_ from C_3_N surface, 6.8 s of recovery time is required. Similarly, a recovery time of 102 s is needed for the desorption of CO from Au-MoS_2_ surface at room temperature [86,87]. The C_2_N surface has been investigated previously by our research group, and very short recovery times of 0.027 s, 0.012 s, and 0.073 s at 298 K for VR, NM, and GF analytes, respectively, had been calculated [25,47]. Desorption results of V-type nerve agents indicates that C_2_N surface can act as a potential candidate as a sensor with suitable recovery time.

## 6. Conclusions

In the present study, the sensing ability of carbon nitride quantum dots (C_2_N) is carried out against V-type nerve agents through DFT at M05-2X/6-31++G(d,p) level of theory. Interaction energy (E_int_) values of optimized geometries predict that all studied V-series@C_2_N complexes are stable (thermodynamically), and adsorption is exothermic. The results indicate that the VE@C_2_N complex is the most stable complex with the highest interaction energy of −17.81 kcal/mol. The stability of the VE@C_2_N complex is attributed due to the presence of strong electrostatic forces, as revealed by QTAIM analysis. Laplacian and electron density values and −V/G ratio of all complexes characterized via QTAIM analysis indicate that only non-covalent interactions exist between V-series and C_2_N units. Non-covalent interaction (NCI) studies depicted by the presence of green spikes revealed weak van der Waals interactions among interacting fragments. FMO analysis indicated that an appreciable decrease in the HOMO-LUMO energy gap (E_H-L_) occurred for all studied complexes. Reduction in band gap also reveals that C_2_N surface is highly sensitive and selective towards V-type nerve agents. A short recovery response time of 3.01 ms at 298 K is predicted for the desorption of VS from C_2_N. The key findings distinctly indicate a better performance of C_2_N surface as an electrochemical sensor towards the VX analyte. We believe that these results will play a crucial role for an experimentalist to tailor a highly selective electrochemical sensor using C_2_N surface.

## Figures and Tables

**Table 1:** Bond lengths (Å) and interaction energies (kcal/mol) of the V-series@C_2_N complexes at the M05-2X/ 6-31++G(d,p) level of theory.

Nitro@C_2_N			
Complex	Intermolecular Bond	Bond Length (Å)	E_int_ (kcal/mol)
VX@C_2_N	H10--N1	2.80	−17.64
	H9--N2	2.75	
	H9--N3	2.38	
	H9--N4	2.85	
	H8--N5	2.40	
	H7--H6	2.78	
VS@C_2_N	H7--N1	2.87	−12.93
	H6--N3	2.96	
	H6--N4	2.84	
	H5--N2	2.99	
VE@C_2_N	H9--N1	2.87	−13.97
	H8--N2	2.55	
	H8--N3	2.72	
	H10--N5	2.72	
	H10--N6	2.65	
	H7--N4	2.72	
VG@C_2_N	H7--N1	2.84	−17.81
	H7--N2	2.40	
	H10--N3	2.42	
	H10--N4	2.72	
	H8--H6	2.90	
	O9--C5	2.84	
VM@C_2_N	H7--N1	2.86	−17.69
	H6--N2	2.79	
	H6--N3	2.91	
	H5--H4	2.66	

**Table 2:** Topological parameters obtained through QTAIM analysis for studied V-series@C_2_N complexes.

Analyte---C_2_N	ρ (a.u)	∇^2^ρ (a.u)	G (r) (a.u)	V(r) (a.u)	H(r) (a.u)	−V/G	E_int_ (kcal/mol)
**VX@C_2_N**							
**H19---C11**	0.007	0.022	0.004	−0.0035	0.0010	0.87	−1.10
**H17---N1**	0.012	0.037	0.008	−0.0077	0.0007	0.96	−2.41
**H20---N10**	0.007	0.022	0.005	−0.0037	0.0010	0.74	−1.16
**H16---N2**	0.006	0.020	0.004	−0.0031	0.0009	0.77	−0.97
**H15---N6**	0.006	0.022	0.004	−0.0035	0.0010	0.87	−1.10
**H15---N3**	0.011	0.036	0.008	−0.0073	0.0009	0.91	−2.29
**H17---N8**	0.006	0.021	0.004	−0.0032	0.0010	0.80	−1.00
**H14---C4**	0.004	0.011	0.002	−0.0017	0.0006	0.85	−0.53
**H13---C5**	0.005	0.017	0.004	−0.0021	0.0008	0.52	−0.66
**S21---C22**	0.008	0.023	0.005	−0.0037	0.0010	0.74	−1.16
**H18---C12**	0.005	0.015	0.003	−0.0023	0.0008	0.77	−0.72
**VS@C_2_N**							
**H20---C10**	0.003	0.012	0.002	−0.0018	0.0006	0.90	−0.56
**H22---N1**	0.004	0.014	0.003	−0.0019	0.0007	0.63	−0.59
**H22---N3**	0.005	0.019	0.004	−0.0029	0.0009	0.72	−0.91
**H20---N12**	0.005	0.018	0.004	−0.0029	0.0008	0.72	−0.91
**H22---N4**	0.005	0.019	0.004	−0.0028	0.0009	0.70	−0.88
**H22---N8**	0.006	0.021	0.004	−0.0032	0.0010	0.80	−1.00
**H22---N11**	0.005	0.019	0.004	−0.0029	0.0009	0.72	−0.91
**H19---C10**	0.004	0.014	0.003	−0.0020	0.0008	0.67	−0.62
**H18---C9**	0.004	0.013	0.003	−0.0018	0.0007	0.60	−0.56
**O16---N8**	0.008	0.026	0.006	−0.0049	0.0009	0.82	−1.54
**H14---C5**	0.005	0.017	0.003	−0.0027	0.0008	0.90	−0.85
**H14---C6**	0.005	0.017	0.003	−0.0027	0.0008	0.90	−0.85
**H21---C13**	0.003	0.012	0.002	−0.0018	0.0006	0.90	−0.56
**O16---N4**	0.009	0.032	0.007	−0.0067	0.0007	0.96	−2.10
**O16---C7**	0.008	0.029	0.006	−0.0055	0.0009	0.92	−1.72
**VE@C_2_N**							
**H9---N3**	0.002	0.008	0.002	−0.0011	0.0005	0.55	−0.34
**H9---N2**	0.002	0.008	0.002	−0.0010	0.0005	0.50	−0.31
**S12---C6**	0.003	0.009	0.002	−0.0011	0.0005	0.55	−0.34
**H10---N4**	0.002	0.008	0.002	−0.0011	0.0005	0.55	−0.34
**H11---N1**	0.003	0.010	0.002	−0.0014	0.0006	0.70	−0.44
**H11---N5**	0.003	0.010	0.002	−0.0014	0.0006	0.70	−0.44
**VG@C_2_N**							
**H12---N10**	0.007	0.025	0.005	−0.0040	0.0010	0.80	−1.25
**O14---C6**	0.004	0.014	0.003	−0.0023	0.0007	0.77	−0.72
**O16---C4**	0.007	0.025	0.005	−0.0040	0.0011	0.80	−1.25
**H13---N1**	0.011	0.033	0.008	−0.0070	0.0007	0.87	−2.19
**H15---N3**	0.011	0.036	0.008	−0.0073	0.0008	0.91	−2.29
**H15---N5**	0.005	0.018	0.004	−0.0030	0.0008	0.75	−0.94
**H13---N2**	0.006	0.020	0.004	−0.0034	0.0008	0.85	−1.07
**H11---C7**	0.006	0.019	0.004	−0.0029	0.0010	0.72	−0.91
**H18---C9**	0.004	0.014	0.003	−0.0021	0.0007	0.70	−0.66
**H17---N8**	0.006	0.020	0.004	−0.0035	0.0008	0.87	−1.10
**H19---N8**	0.004	0.014	0.003	−0.0023	0.0006	0.77	−0.72
**VM@C_2_N**							
**O21---C6**	0.005	0.018	0.004	−0.0031	0.0008	0.77	−0.97
**H16---N12**	0.005	0.017	0.003	−0.0025	0.0008	0.83	−0.78
**O21---C4**	0.005	0.017	0.004	−0.0029	0.0007	0.72	−0.91
**O21---C5**	0.005	0.018	0.004	−0.0031	0.0007	0.77	−0.97
**O20---N10**	0.009	0.029	0.007	−0.0060	0.0006	0.86	−1.88
**H17---C13**	0.007	0.023	0.005	−0.0037	0.0010	0.74	−1.16
**H19---C11**	0.004	0.014	0.003	−0.0020	0.0008	0.67	−0.62
**H16---N2**	0.006	0.019	0.004	−0.0029	0.0010	0.72	−0.91
**H16---N3**	0.006	0.020	0.004	−0.0031	0.0010	0.77	−0.97
**H16---N7**	0.005	0.016	0.003	−0.0024	0.0008	0.80	−0.75
**H16---N10**	0.004	0.014	0.003	−0.0020	0.0007	0.67	−0.63
**H15---N1**	0.005	0.018	0.004	−0.0029	0.0008	0.72	−0.91
**H23---N9**	0.004	0.015	0.003	−0.0024	0.0007	0.80	−0.75

**Table 3:** SAPT0 analysis of V-series@C_2_N complexes (kcal/mol).

Complexes	∆E_elst_	%	∆E_exch_	∆E_ind_	%	∆E_dis_	%	∆E_SAPT0_
VX@C_2_N	−13.18	26.60	23.28	−4.49	9.06	−31.88	64.34	−26.27
VS@C_2_N	−10.28	21.24	22.20	−5.12	10.59	−32.99	68.17	−26.20
VE@C_2_N	−10.66	26.18	18.23	−3.47	8.52	−26.58	65.29	−22.49
VG@C_2_N	−15.39	29.20	25.14	−5.83	11.06	−31.48	59.73	−27.56
VM@C_2_N	−9.37	21.39	19.75	−4.62	10.55	−29.80	68.05	−24.04

**Table 4:** FMO and NBO results of most stable V-series@C_2_N complexes.

Complexes	HOMO eV	LUMO eV	E_H-L_	NBO (e^−^)
**VX@C_2_N**	−5.02	−2.89	2.13	−0.023
**VS@C_2_N**	−5.37	−2.74	2.63	−0.020
**VE@C_2_N**	−5.35	−2.88	2.47	−0.012
**VG@C_2_N**	−5.56	−2.75	2.81	−0.013
**VM@C_2_N**	−5.57	−2.78	2.80	−0.002
**C_2_N**	−6.40	−2.69	3.71	--
